# Prognostic prediction of hypertensive intracerebral hemorrhage using CT radiomics and machine learning

**DOI:** 10.1002/brb3.2085

**Published:** 2021-02-24

**Authors:** Xinghua Xu, Jiashu Zhang, Kai Yang, Qun Wang, Xiaolei Chen, Bainan Xu

**Affiliations:** ^1^ Department of Neurosurgery The First Medical Center of Chinese PLA General Hospital Beijing China; ^2^ Department of Neurosurgery Dongying People's Hospital Dongying China

**Keywords:** artificial intelligence, intracerebral hemorrhage, machine learning, prognosis, radiomics

## Abstract

**Objectives:**

Spontaneous intracerebral hemorrhage remains a major cause of death and disability throughout the world. We tried to establish accurate long‐term outcome prediction models for hypertensive intracerebral hemorrhage (HICH) using CT radiomics and machine learning.

**Methods:**

In a retrospective study of 270 patients with HICH between June 2013 and June 2018, CT images and patients' 6‐month outcome based on the modified Rankin Scale were collected. Hematomas on CT images were selected as volumes of interests (VOIs), and 1,029 radiomics features of the VOIs were extracted. Based on correlations with patients' outcome, radiomics features underwent dimensionality reduction analyses. Then, the support vector machine (SVM), k‐nearest neighbor (KNN), logistic regression (LR), decision tree (DT), random forest (RF), and XGBoost algorithms were applied with the screened features to establish prognostic prediction models of HICH. Accuracies of all models were compared.

**Results:**

Eighteen radiomics features were screened as prognosis‐associated radiomics signature of HICH based on the variance threshold, SelectKBest, and least absolute shrinkage and selection operator (LASSO) regression models. Patients were randomly allocated into training (*n* = 215) and validation (*n* = 55) sets. Accuracies of all 6 machine learning algorithms in the validation set exceeded 80%. The sensitivity, specificity, and accuracy in the validation set were 93.3%, 92.5%, and 92.7% for the RF model and 92.3%, 88.1%, and 89.1% for the XGBoost model, respectively, which were the best two among all models.

**Conclusions:**

Taking advantage of radiomics and machine learning, we established accurate prognostic prediction models of HICH. The RF model and XGBoost model returned the best accuracies.

## Graphical abstract

We tried to establish accurate long‐term outcome predictions models for hypertensive ICH using CT radiomics and machine learning. Six machine learning algorithms were used at the same time, and the accuracies of different models were compared and selected. Finally, the random forest algorithm model demonstrated a sensitivity of 93.3%, a specificity of 92.5% and the area under the curve was 0.92, which were the best among all 6 models.

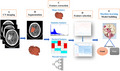

## 1 INTRODUCTION

Spontaneous intracerebral hemorrhage (ICH) is the most devastating stroke type, with reported 30‐day mortality rate being as high as 40%, and only one‐fifth of survivors can live independently 6 months after ictus (van Asch et al., 2010). Hypertensive ICH (HICH) is the most common type of ICH, accounting for approximately 70% of all ICHs (Meretoja et al., 2012). Studies have identified a wide range of factors associated with outcome after acute ICH. Identification of these factors led to the development of models to predict mortality and functional outcome (Ariesen et al., 2005; Cheung & Zou, 2003; Rost et al., 2008; Ruiz‐Sandoval et al., 2007). Early prognostication is often desired by doctors, patients, and families, but existing prognostic models are biased and no models have satisfactory accuracy (Hemphill et al., 2015).

The rapid development and advancement of medical imaging technology has provided more comprehensive data and has played an increasingly important role in disease screening, treatment planning, and prognosis assessment (Doi, 2007). Biomedical images contain information that reflects underlying pathophysiology and these relationships can be revealed via high‐throughput quantitative image analyses (Gillies et al., 2016). The process of converting digital medical images into mineable high‐dimensional data is known as radiomics (Lambin et al., 2012). Machine learning is a field of computer science that uses statistical techniques to give computer systems the ability to “learn” with data without being explicitly programmed (Deo, 2015; Jordan & Mitchell, 2015). In recent years, machine learning algorithms have been applied to cancer research to predict genotype preoperatively or to predict patients' prognosis based on radiomics features (Emblem et al., 2015; Lu et al., 2018; Macyszyn et al., 2015; Zhang et al., 2017). In this study, we attempted to establish accurate long‐term outcome prediction models for HICH using CT radiomics and machine learning algorithms.

## 2 MATERIALS & METHODS

### 2.1 Patients

Between June 2013 and June 2018, 270 patients hospitalized for HICH were included in the study (80 women, 190 men; mean age 54.7 ± 11.2 years). Inclusion criteria were as follows: (a) confirmed diagnosis of supratentorial HICH; (b) available CT images scanned within 24 hr after hemorrhage; (c) available prognosis information at 6 months after ictus based on the modified Rankin Scale (mRS); (d) clinical characteristics (i.e., age, gender, and hemorrhage location) available. Prognosis was dichotomized according to the mRS: Good outcome was defined as mRS <3, whereas poor outcome was defined as mRS ≥3. This retrospective study was approved by the Chinese PLA General Hospital Institutional Review Board (IRB), and verbal agreement was obtained.

### 2.2 Image acquisition, segmentation, and radiomics features extraction

A noncontrast CT scan was performed on all patients after admission to the hospital and the CT image data were collected in the format of Digital Imaging and Communications in Medicine (DICOM) so as to contain as much original information as possible. Hematoma outlines on CT images were regarded as volume of interests (VOIs) and were drawn slice‐by‐slice semi‐automatically by a neurosurgeon in order to establish a precise relationship between hematoma radiomics features and prognosis.

Radiomics features extracted from each VOI were calculated automatically. The features could be divided into 5 groups: (a) first‐order statistic, (b) shape features, (c) gray‐level co‐occurrence matrix (GLCM), (d) gray‐level run length method (GLRLM), and (e) gray‐level size zone matrix (GLSZM). The final set consisted of 19 first‐order statistic, 15 shape and size features, and 59 textural features (including 27 GLCM, 16 GLRLM, and 16 GLSZM). The first‐order statistic features and textural features underwent 12 types of transformation and filter: exponential, square, square root, logarithm, and wavelets (wavelet‐LLL, wavelet‐HHH, wavelet‐HLL, wavelet‐HHL, wavelet‐LLH, wavelet‐HLH, wavelet‐LHL, wavelet‐LHH). Finally, a total of 1,029 [(19 + 59) × 12 + 19+59 + 15] radiomics features were extracted from each patient's CT images.

### 2.3 Dimensionality reduction and analysis of radiomics features

The principal features relevant for prognosis were then identified by reducing the number of features under consideration. Three feature selection methods, variance threshold, SelectKBest, and the least absolute shrinkage and selection operator (LASSO) were applied orderly to screen out the features that could best predict prognosis of HICH. The variance threshold was applied to evaluate the divergence, and features with variance less than 0.8 were abandoned since only divergent features could play a part in differentiating outcome. SelectKBest was a univariate feature selection method that used variance analysis to measure the relationship between features and outcome. LASSO was a regression analysis method that performed both feature selection and regularization in order to enhance the prediction accuracy and interpretability of the statistical model it produces.

To further remove irrelevant or redundant features to improve data quality and speed up data analysis, principal component analysis (PCA), covariance analysis, and cluster analysis were performed in turn. Principal component analysis used the orthogonal transformation to convert possibly correlated features into a set of linearly uncorrelated features to evaluate the correlation between these features. Covariance analysis was used to assess the degree to which two selected features cooperated or interacted. Cluster analysis grouped a set of features in a way that features in the same group were more similar to each other than to those in other groups.

### 2.4 Machine learning and prognosis prediction

**FIGURE 1:**
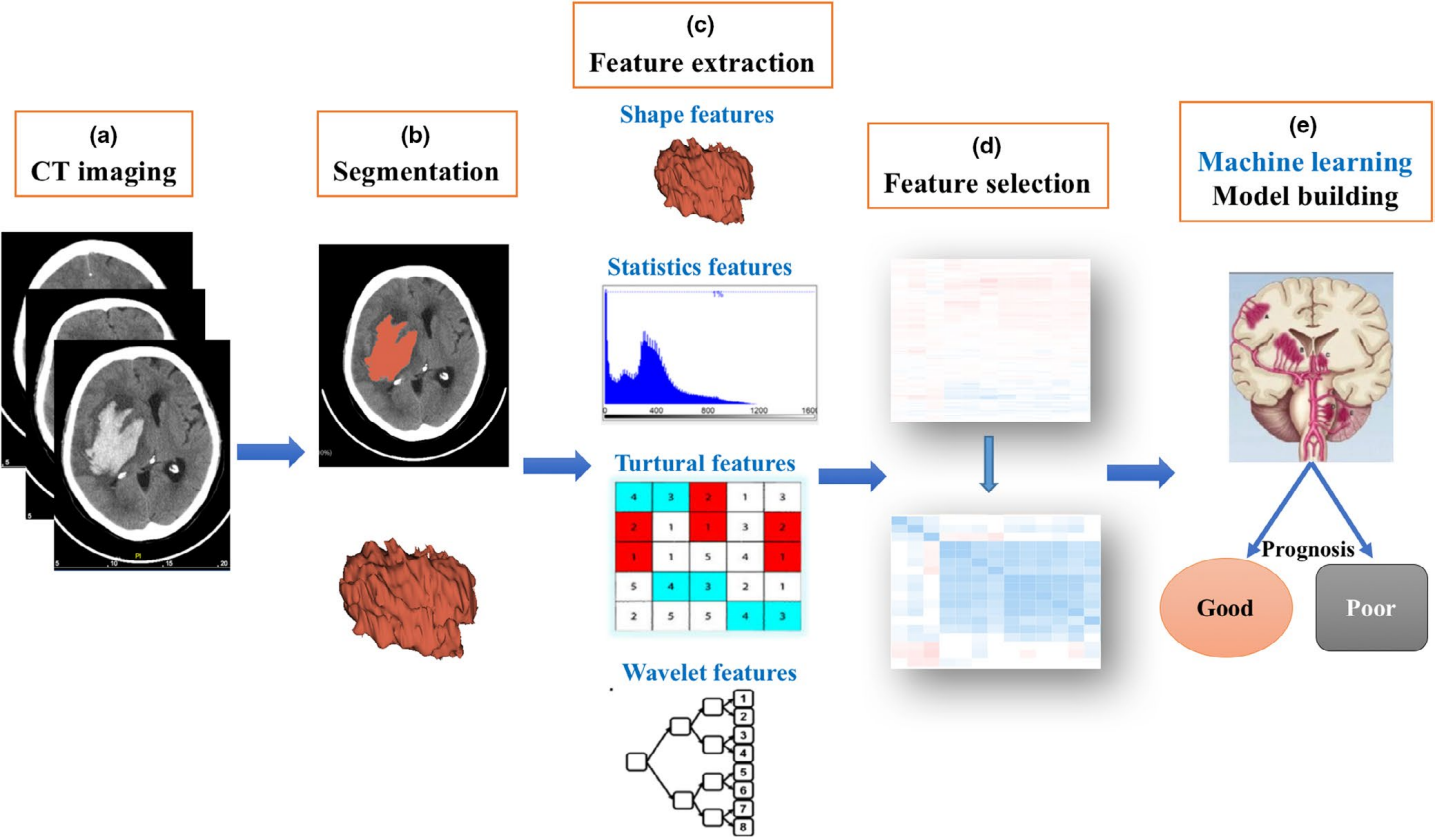
The image postprocessing workflow. First, hematoma on CT images was segmented. After feature extraction, feature selection, and machine learning model construction, six prognosis‐predictive models were established in the training set and were further evaluated in the validation sets

Six common machine learning algorithms, support vector machine (SVM), k‐nearest neighbor (KNN), logistic regression (LR), decision tree (DT), extreme gradient boosting (XGBoost), and random forest (RF) were used to establish a prediction model of prognosis after HICH based on the selected features. The SVM aims to create a decision boundary between two classes that enables the prediction of labels from one or more feature vectors. KNN algorithm is a nonparametric approach used for classification, and it customs the information about its neighbor points for the classification of output labels. Logistic regression iteratively identifies the strongest linear combination of variables with the greatest probability of detecting the observed outcome using components of linear regression reflected in the logit scale. Decision tree denotes a tree with its node refers to the attribute, whereas its link refers to a decision rule and its leaf node refers to an output class. Extreme gradient boosting (XGboost) is an improved supervised learning algorithm based on the Gradient Boosting Decision Tree algorithm. The Random Forest technique is a regression tree technique, which uses bootstrap aggregation and randomization of predictors to achieve a high degree of predictive accuracy. Of the 270 HICH patients, 80%, 215 patients, were used as the training set to train the machine learning prognosis prediction models and the remaining 55 patients (20%) were used as the test set to evaluate the performance of the prediction models. Sensitivity, specificity, and accuracy in training set and test set were calculated to quantify their prediction performance. The receiver operating characteristic (ROC) curve was presented, and the area under the curve (AUC) represented the prediction power of a classifier was reported. A larger AUC indicated a better prediction power. An accuracy close to 1 in the training set represented overfitting, which was the production of an analysis that corresponded too closely or exactly to a particular set of data but failed to fit additional data reliably. The image data processing and machine learning workflow are shown in Figure 1.

### 2.5 Statistical analysis

All statistical analyses were performed in Radcloud platform. Computer‐generated random numbers were used to assign 80% of the VOIs to the training data set and 20% of VOIs to the validation data set.

## 3 RESULTS

### 3.1 Clinical characteristics

A total of 270 supratentorial HICH patients with 6‐month follow‐up data were included in this study. There were more male patients (70.4%) than female patients. At 6 months of follow‐up using the mRS score, 87 patients (32.2%) had good outcome (mRS <3) and 183 patients (67.8%) had poor outcome. The overall prognosis of patients with HICH was poor.

### 3.2 Feature extraction, selection, and analysis

**FIGURE 2:**
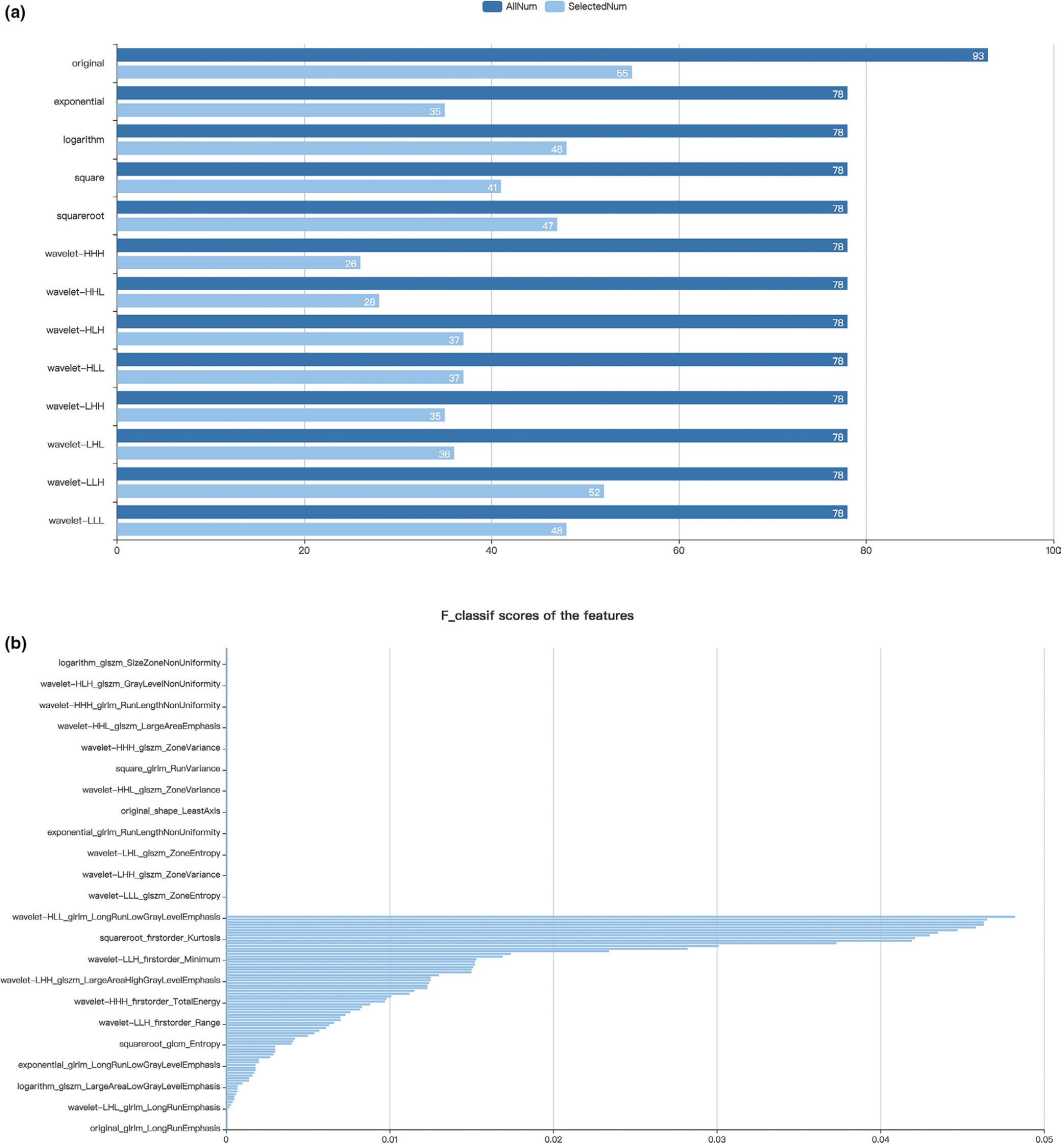
Figure selection using variance threshold analysis and SelectKBest analysis. Of the 1,029 features extracted, 525 features were selected after variance threshold analysis (a) and 182 were selected after SelectKBest analysis (b)

**FIGURE 3:**
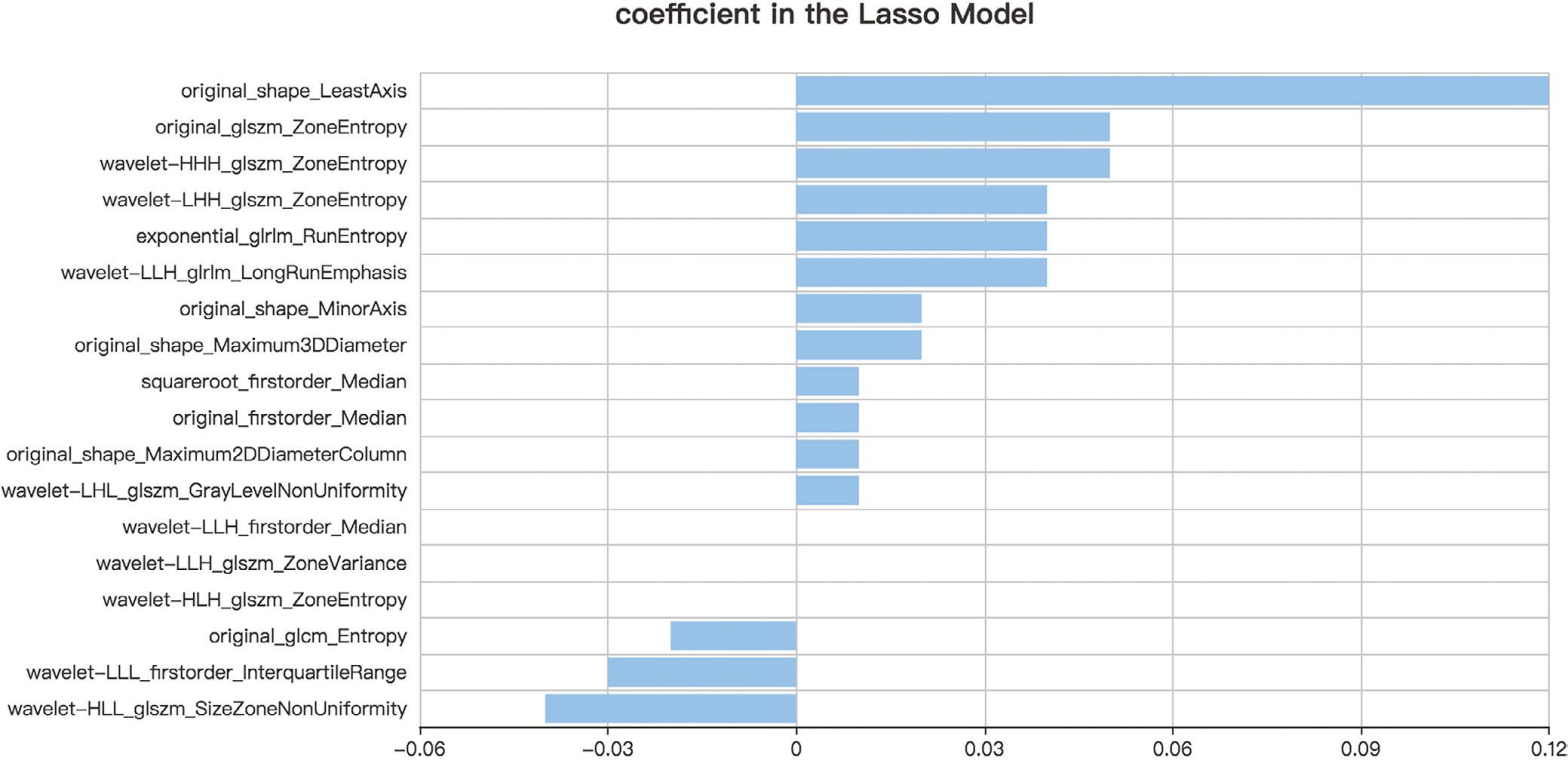
Features finally selected for prediction after the LASSO regression analysis

**FIGURE 4:**
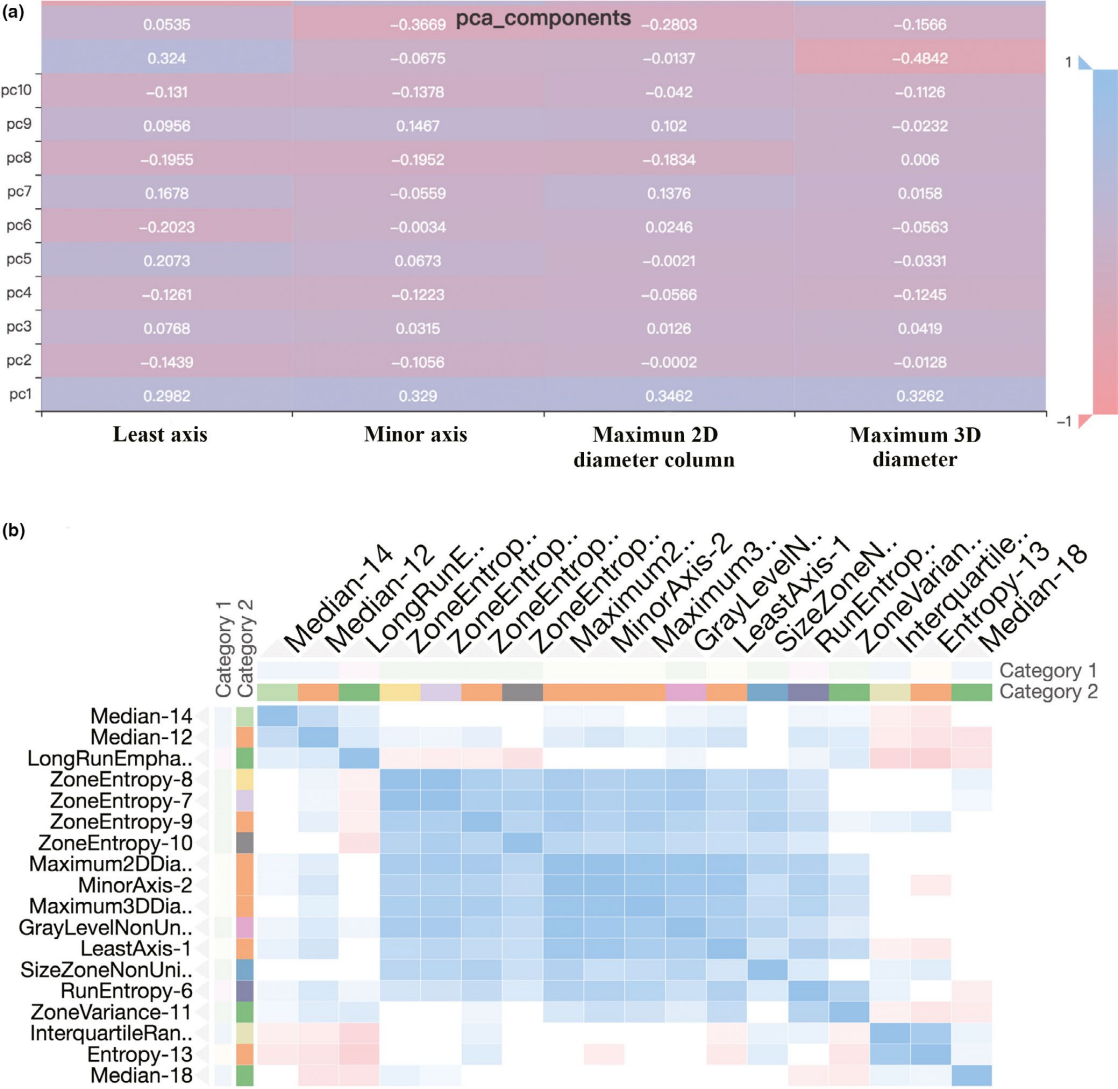
Principal component screening and feature correlation analysis. Principal component analysis revealed the 4 features that had the greatest impact on prognosis (a). Covariance analysis showed the interdependence and relevance between the selected 18 features (b)

First, the 1,029 radiomics features of all patients were extracted. Then, the extracted 1,029 original radiomics features were reduced to 525 and then to 182 after variance threshold analysis and SelectKBest analysis (Figure 2). Finally, 18 radiomics features were nonzero coefficients after the LASSO regression analysis. Details of the selected 18 features were shown in Figure 3. PCA showed that the features that contributed most to patients' prognosis were as follows: least axis, minor axis, maximum 2D diameter column, and maximum 3D diameter (Figure 4a). Covariance analysis showed the interdependence and collaborative changes between the selected 18 radiomics features (Figure 4b), and cluster analysis revealed the distribution of the selected features in all 270 patients.

### 3.3 Machine learning established prediction models

**FIGURE 5:**
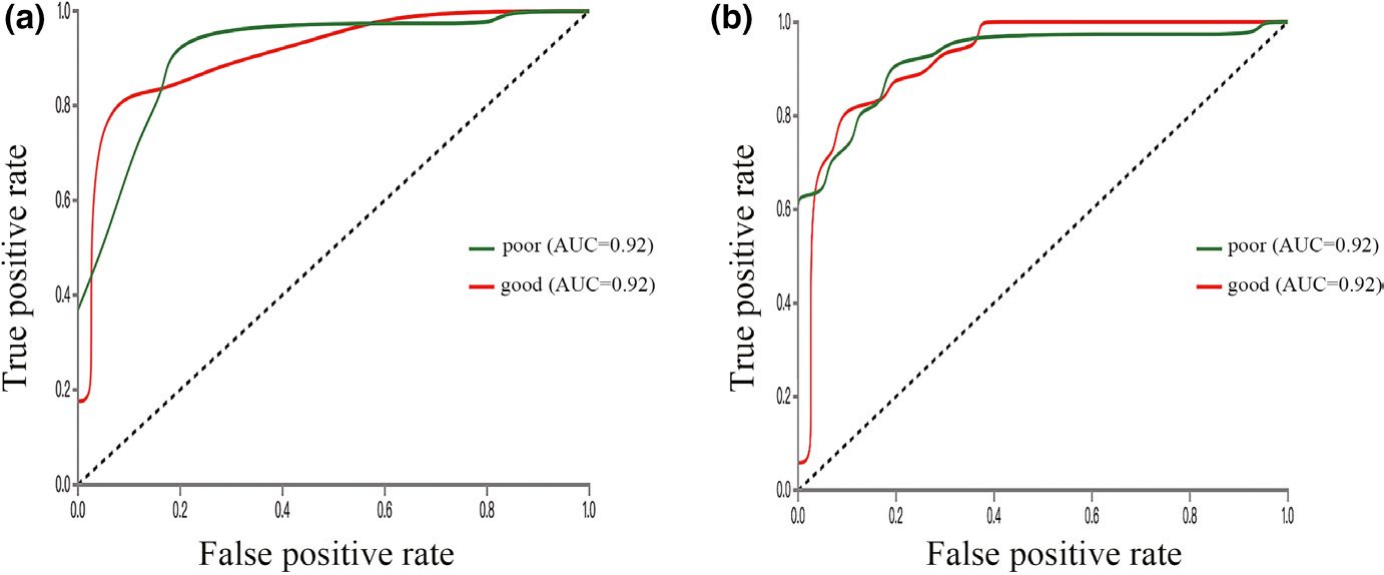
ROC curves of the RF model and the XGBoost model for long‐term outcome prediction of HICH in validation sets. The AUC was 0.92 for both the RF model (a) and the XGBoost model (b)

The sensitivity, specificity, and overall accuracy of all 6 prediction models established by different machine learning algorithms for training set and validation set were summarized in Table 1. All 6 algorithms could distinguish good outcome HICH patients from poor outcome patients accurately. General accuracies of the DT algorithm model were 100% in the training set and 85.5% in the validation set, which suggested overfitting. The RF and XGBoost were the two most accurate algorithms for both training set and validation set. The sensitivity, specificity, and accuracy in the validation set were 93.3%, 92.5%, and 92.7% for the RF model and 92.3%, 88.1%, and 89.1% for the XGBoost model, respectively. Fifty‐one out of 55 HICH patients in the validation set were predicted correctly according to the RF prediction model, and 49 out of 55 were predicted correctly according to the XGBoost prediction model. The AUC was 0.92 (95% confidence interval [95% CI], 0.82–0.97) for the RF model and 0.92 (95% CI, 0.74–0.93) for the XGBoost model (Figure 5). Even though the overall accuracy of the KNN prediction model in the validation set (83.6%, 46/55) was the lowest among all 6 models, the accuracy was still higher than 80%. CT radiomics‐based machine learning algorithms, especially the RF algorithm and the XGBoost algorithm, could accurately predict 6‐month outcome in patients with supratentorial HICH. Based on the above findings, intelligent prediction of prognosis after supratentorial HICH was possible.

**TABLE 1:** Sensitivity, specificity, and overall accuracy of different machine learning algorithm prediction models for training set and validation set

Algorithms	Training set (*n* = 215)			Validation set (*n* = 55)		
	Sensitivity	Specificity	Accuracy	Sensitivity	Specificity	Accuracy
KNN	86.8%	88.3%	87.9%	90.0%	82.2%	83.6%
SVM	81.8%	87.5%	86.0%	90.9%	84.1%	85.5%
XGBoost	96.0%	89.7%	91.2%	92.3%	88.1%	89.1%
RF	98.5%	99.3%	99.1%	93.3%	92.5%	92.7%
LR	75.9%	86.6%	83.7%	90.9%	84.1%	85.5%
DT	100%	100%	100%	80.0%	87.5%	85.5%

Abbreviations: DT, decision tree; KNN, k‐nearest neighbor; LR, logistic regression; RF, random forest; SVM, support vector machine; XGBoost, extreme gradient boosting.

## 4 DISCUSSION

Spontaneous ICH is a type of stroke with the highest mortality and disability rate, and its mortality decreased little in the past 30 years (van Asch et al., 2010; Vibo et al., 2007). Regardless of the choice of treatment, prognosis is what doctors, families, and patients care about the most. Previous studies have identified a wide range of factors associated with worse outcome after HICH, including older age, lower Glasgow Coma Scale (GCS) score, larger hematoma volume, combined intraventricular hemorrhage, and deep or infratentorial hemorrhage. Identification of these factors led to the development of models for predicting death or functional outcome after ICH, such as the FUNC score, the NIH‐SS, and the intracerebral hemorrhage score (Ariesen et al., 2005; Cheung & Zou, 2003; Garrett et al., 2013; Rost et al., 2008; Ruiz‐Sandoval et al., 2007; Weimar et al., 2006). Cheung & Zou reported that the ICH score compromising the GCS score, age, infratentorial origin, ICH volume, and intraventricular hemorrhage served to predict good outcome, with a sensitivity of 93.5%, and a specificity of 60.5% in a cohort of 142 patients (Cheung & Zou, 2003). Ruiz‐Sandoval et al reported an ICH grading scale with sensitivity of 70.0%, and specificity of 86.7% for 30‐day good outcome in 310 patients (Ruiz‐Sandoval et al., 2007). On the whole, previously reported prediction methods focused on short‐term prognosis and have relatively low accuracies. In addition, the assessment of hematoma volume using the Tada formula was rough and inaccurate (Xu et al., 2014).

In this study, we presented a new method to predict the outcome of HICH. The primary objective of our study was to establish an accurate prognostic prediction model for HICH by using CT radiomics and machine learning. High‐throughput radiomics of 1,029 quantitative CT features were extracted to assess their value in predicting prognosis of HICH. Finally, 18 radiomics features were screened out as imaging markers to establish prediction models using machine learning algorithms. Through 6 advanced machine learning algorithms, we not only established 6 HICH prognosis prediction models, but also evaluated and verified the accuracy of each prediction model. Compared with traditional visual image assessment, radiomics could obtain more comprehensive information. The sensitivity, specificity, and accuracy of the RF algorithm prediction model all exceeded 90% in the validation set, which were significantly higher than previously reported methods. Our findings demonstrated that CT radiomics and machine learning‐based prediction models could accurately predict the 6‐month outcome in patients with supratentorial HICH.

Radiomics aims to extract large amount of quantitative features from medical images using data‐characterization algorithms (Kumar et al., 2012; Lambin et al., 2012). These features, namely radiomics features, have the potential to uncover disease characteristics that fail to be appreciated by the naked eye (Aerts et al., 2014; Wu et al., 2018). Radiomics features include metrics such as spatial relationships, textural heterogeneity, and many other characteristics, and the distinctive imaging features between disease forms may be useful for predicting prognosis (Lambin et al., 2012; Yip & Aerts, 2016). It has been proven that radiomics is able to accurately predict genotype, gene mutation status, and survival in tumors, such as isocitrate dehydrogenase (IDH) genotype, O_6_‐methylguanine‐DNA methyltransferase promoter methylation, 1p19q codeletion, and p53 (Emblem et al., 2015; Jakola et al., 2018; Li et al., 2017; Xi et al., 2018). Zhang et al generated a model predictive of IDH genotype in high‐grade gliomas with accuracies of 86% (AUC = 0.88) in the training cohort and 89% (AUC = 0.92) in the validation cohort using random forest algorithm (Zhang et al., 2017). In another similar study, the authors generated a model predictive of IDH mutation status achieving an AUC of 0.921 in the training cohort and 0.919 in the validation cohort and a model predictive of 1p19q codeletion achieving an 0.917 and 0.916 for the training and validation cohort (Zhou et al., 2019). Lu et al reported the IDH and 1p/19q status of gliomas could be classified by radiomics and machine learning approaches, with AUC between 0.922 and 0.975 and accuracies between 87.7% and 96.1%. Emblem et al developed a support vector machine model with high diagnostic accuracies for 6‐month and 1‐, 2‐, and 3‐year survival (AUC 0.794–0.851) (Emblem et al., 2015). On the whole, current radiomics and machine learning‐related researches mainly focused on prediction of genotype or survival of tumors. To our knowledge, our study was a new attempt to predict prognosis in HICH patients and we got favorable accuracies similar to those in tumor‐related studies.

Before the advent of radiomics studies, several CT image markers or signs have proven to significantly correlate with hemorrhage expansion and patients' prognosis, such as hemorrhage volume, hemorrhage margin irregularity, black hole sign, hypodensity, and density heterogeneity (Blacquiere et al., 2015; Boulouis et al., 2016; Brouwers et al., 2014; Delcourt et al., 2016; Li et al., 2016). Radiomics can be regarded as quantification and full extension of above mentioned features and signs. Machine learning offers an approach for discovering predictive radiomics features and establish prediction model (Zhou et al., 2018). The parameter space is searched for imaging features statistically associated with clinical outcome. The results demonstrated that the deep features extracted via machine learning performed much better than traditional image signs seen by the naked eye in the prediction of long‐term outcome in HICH patients.

The results of our study showed that 18 of 1,029 radiomics features were most closely associated with prognosis of HICH, including 4 first‐order statistic features, 4 shape features, and 10 textural features. Most of these features were not visually appreciable but very important for comprehensive assessment of patient's state and prediction of the long‐term outcome. We got a higher accuracy compared with the aforementioned studies concerning tumor survival and gene mutation status using radiomics and machine learning (Emblem et al., 2015; Jakola et al., 2018; Li et al., 2017; Xi et al., 2018). The reason might be that we used 6 different machine learning algorithms to establish 6 prediction models at the same time and chose the one with the highest accuracy. Machine learning by means of RF or XGBoost made the early prediction of long‐term outcome after HICH possible and could be used for artificial intelligence prediction of HICH prognosis.

Despite the promising results, there are several limitations in our study. First, the imaging data were not acquired from the same CT scanner, which might contribute to model performance discrepancy. In addition, different treatment measures patients received might be a confounding factor for predicting outcomes. Finally, limited by the retrospective nature of our study, a prospective study with more patients is warranted to verify the results.

## 5 CONCLUSIONS

In this study, we established 6 long‐term outcome prediction models for HICH using radiomics and machine learning algorithms. After comparison, the RF model and the XGBoost model showed the best accuracy and are attractive alternatives to traditional methods for upfront assessment of long‐term outcome in supratentorial HICH patients.

## CONFLICT OF INTEREST

All authors have no conflicts of interest to disclose.

## AUTHOR CONTRIBUTION

Xinghua Xu performed conceptualization and design of the study, interpretation of data, and drafting and revising the manuscript. Jiashu Zhang involved in design of the study, acquisition of data, and interpretation of data. Kai Yang designed the study and interpreted the data. Qun Wang collected and analyzed the data. Xiaolei Chen critically revised the manuscript, supervised the study, and supported the funding. Bainan Xu supervised the study and supported the funding. All authors have read and approved the manuscript.

## Data Availability

The data that support the findings of this study are available from the corresponding author upon reasonable request.
